# Mechanical stimulation promotes MSCs healing the lesion of intervertebral disc annulus fibrosus

**DOI:** 10.3389/fbioe.2023.1137199

**Published:** 2023-02-10

**Authors:** Rongrong Deng, Ran Kang, Xiaoyu Jin, Zihan Wang, Xin Liu, Qing Wang, Lin Xie

**Affiliations:** ^1^ Third School of Clinical Medicine, Nanjing University of Chinese Medicine, Nanjing, Jiangsu, China; ^2^ Department of Orthopedics, Nanjing Lishui Hospital of Traditional Chinese Medicine, Nanjing, Jiangsu, China; ^3^ Department of Sports Medicine and Adult Reconstructive Surgery, Nanjing Drum Tower Hospital, Nanjing, Jiangsu, China; ^4^ School of Nursing, Nanjing University of Chinese Medicine, Nanjing, Jiangsu, China

**Keywords:** mesenchymal stem cells, gel scaffold, intervertebral disc annulus fibrosus, mechanical stimulation, RhoA/Rock1 pathway

## Abstract

Mesenchymal stem cells (MSCs) and scaffolds offer promising perspectives for annulus fibrosus (AF) repair. The repair effect was linked to features of the local mechanical environment related to the differentiation of MSCs. In this study, we established a Fibrinogen-Thrombin-Genipin (Fib-T-G) gel which is sticky and could transfer strain force from AF tissue to the human mesenchymal stem cells (hMSCs) embedded in the gel. After the Fib-T-G biological gel was injected into the AF fissures, the histology scores of intervertebral disc (IVD) and AF tissue showed that Fib-T-G gel could better repair the AF fissure in caudal IVD of rats, and increase the expression of AF-related proteins including Collagen 1 (COL1), Collagen 2 (COL2) as well as mechanotransduction-related proteins including RhoA and ROCK1. To clarify the mechanism that sticky Fib-T-G gel induces the healing of AF fissures and the differentiation of hMSCs, we further investigated the differentiation of hMSCs under mechanical strain *in vitro*. It was demonstrated that both AF-specific genes, including Mohawk and SOX-9, and ECM markers (COL1, COL2, aggrecan) of hMSCs were up-regulated in the environment of strain force. Moreover, RhoA/ROCK1 proteins were also found to be significantly up-regulated. In addition, we further -demonstrated that the fibrochondroinductive effect of the mechanical microenvironment process could be significantly blocked or up-regulated by inhibiting the RhoA/ROCK1 pathway or overexpressing RhoA in MSCs, respectively. Summarily, this study will provide a therapeutic alternative to repair AF tears and provide evidence that RhoA/ROCK1 is vital for hMSCs response to mechanical strain and AF-like differentiation.

## 1 Introduction

Mechanical strain stimulates the development and remodeling of the intervertebral disc (IVD) from mesodermal cells during human body growth and development (Sivakamasundari and Lufkin, 2012; Colombier et al., 2014; Duclos and Michalek, 2017). IVD is structured by the surrounding highly oriented collagenous annulus fibrosus (AF), the central gelatinous nucleus pulposus, and the craniocaudal endplate (Marchand and Ahmed, 1990; Chan et al., 2011b). The integrity of IVD is secured the spine stability to withstand a load of tensile, compression, and shear in our daily life (Schmidt et al., 2007; Chan et al., 2011a). However, disc lesion occurs inevitably along with aging, load fatigue, trauma, etc. (Battié et al., 2007; Wang et al., 2016), resulting in painful disc herniation, reherniation, and degeneration. Repair is necessary, especially for AF, because anatomically the outer AF holds the central nucleus material and prevents disc herniation and degeneration (Holzapfel et al., 2004).

Suturing, anchoring (Sharifi et al., 2015), and tissue engineering (Bron et al., 2008; Kang et al., 2018) (Chu et al., 2018b; Shamsah et al., 2020)are found to help heal AF lesions, though they are still far from clinical application. The imperfect results may be due to stress shielding by these physical repair methods in the lesion region (Chu et al., 2018a). Mechanically-stimulated tissue regeneration is gaining more attention in musculoskeletal tissue repair strategies. Since extracellular matrix (ECM) components impact stem cell differentiation (Smith et al., 2018; MacDonald et al., 2021), a repair method utilizing biological glue could connect tissue lesions and the microenvironment could transfer strain stress to the immersed seeding cells, such as mesenchymal stem cells (MSCs), to gain more successful differentiation and repair effects (Kang et al., 2017).

MSCs differentiation under proper mechanical stimulation already successfully generates quality cartilage, tendon, bone, and cardiomyogenic tissue (Gu et al., 2017; Kataoka et al., 2020; Liu et al., 2022a; Park et al., 2022; Volz et al., 2022). Recently, the frequency and intensity of mechanical stimulation have been optimized in several studies (Chan et al., 2011a; Lee et al., 2016; Elsaadany et al., 2017). Studies also revealed that the mechanical-related RhoA/ROCK signaling pathway regulation might be the intrinsic mechanism for such a phenomenon (Xu et al., 2012; Peng et al., 2021). More specifically, for AF repair, the tensile stress of 1 HZ frequency and 3% magnitude has been reported to be the favorite mechanical stimulation for MSCs differentiation to AF *in vitro* (Elsaadany et al., 2017). However, few studies specifically compare the repair effect of AF lesions under mechanical shielding and mechanical stimulation, since there is still a lack of a proper biological glue with ideal biocompatibility and sufficient adhesive strength to repair the AF defect.

**FIGURE 1:**
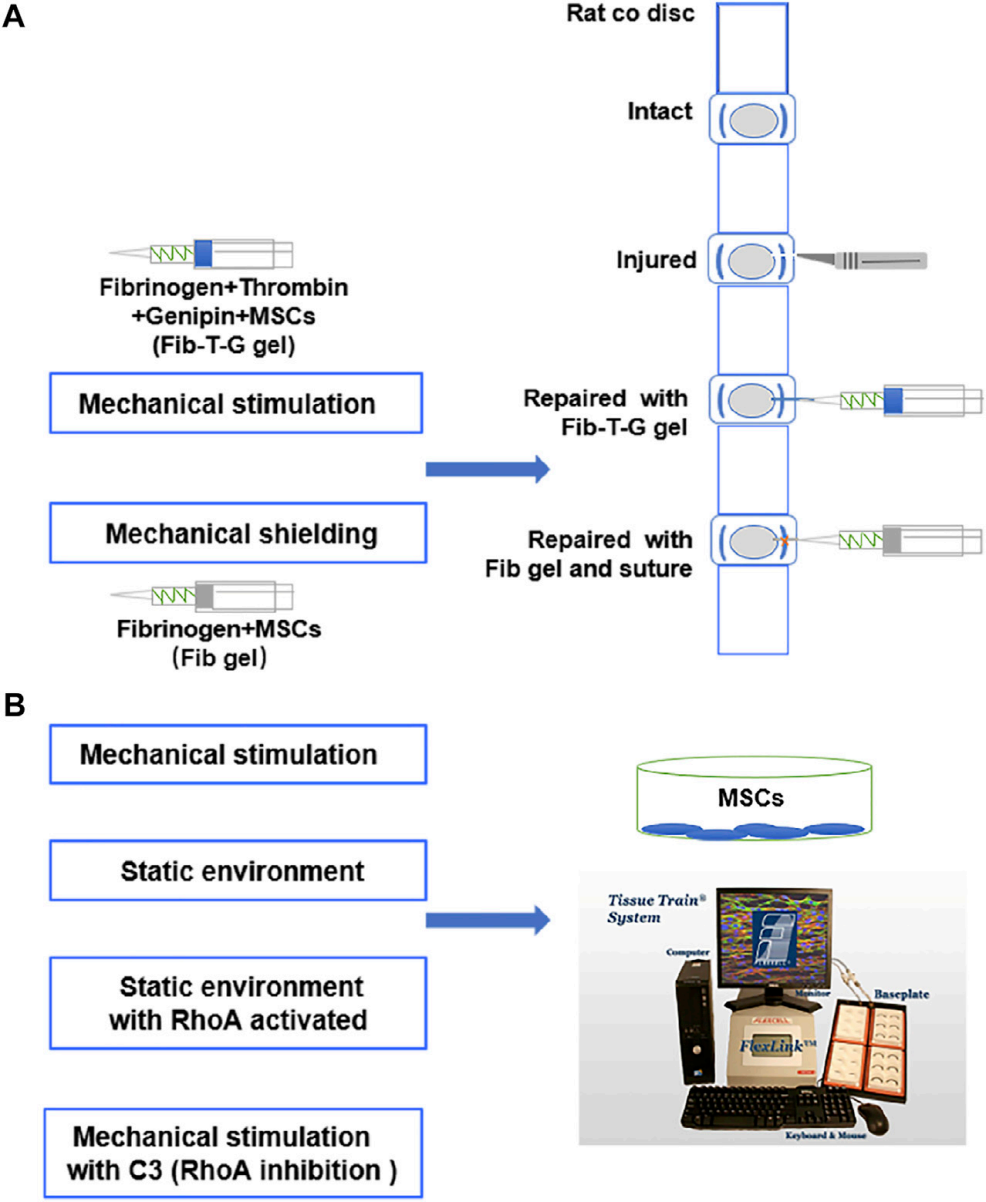
**(A)** Schematic diagram of rat annulus fibrosus injury repair by injectable Fibrinogen-Thrombin-Genipin (Fib-T-G) gel mixed MSCs. **(B)** Schematic diagram of the intrinsic molecular mechanism of MSCs differentiation into annulus fibrosus *in vitro* mechanical stimulation environment.

We established a biological glue composed of Fibrinogen-Thrombin-Genipin (Fib-T-G), which is competent for AF defect repair in a rat model in our pilot study. We further designed the study and investigated the repair quality *in vivo* under two different mechanical conditions: a mechanical stimulation environment by human MSCs (hMSCs) mixed in Fib-T-G sticky glue to fill and connect the annulus lesion; a mechanical shielding environment by hMSCs mixed in Fibrinogen non-sticky solution to fill, and together with suture to connect the annulus lesion. We also studied the molecular reactions of hMSCs in a mechanical stimulation environment as well as the possible intrinsic molecular mechanism of the differentiation of hMSCs towards AF tissue *in vitro* (Figure 1).

## 2 Materials and methods

### 2.1 Fib-T-G gel and MSCs preparation

Fibrinogen (F3879, Sigma, United States) dissolved in phosphate-buffered saline (PBS, 0.01 M, pH 7.40) at a concentration of 200 mg/ml, thrombin (T4393, Sigma, United States) dissolved in PBS at a concentration of 100 U/ml, and genipin (G4796, Sigma, United States) dissolved in dimethyl sulfoxide (D2650, Sigma, United States) at a concentration of 400 mg/ml, were prepared for the glue fabrication. This component concentration of Fib-T-G gel was optimized by testing the biocompatibility and mechanical properties with different concentrations of genipin. The Fib-T-G gel contained final concentrations of fibrinogen 140 mg/ml, thrombin 28 U/ml, and genipin 6 mg/ml–these concentrations exhibited the best consistency for biocompatibility and adhesive strength for the following experiment in our pilot study, which was added as Supplementary Data. hMSCs (Passage 2, 15901; purchased from ScienCell Research Laboratories, Carlsbad, CA) routine cultured in Mesenchymal Stem Cell medium (7501, Sciencell, United States) which included DMEM supplemented with 5% fetal bovine serum (FBS, 0025, Sciencell, United States), 1% penicillin-streptomycin (PS, 0503, Sciencell, United States) and Mesenchymal Stem Cell Growth Supplement (MSCGS) (7552, Sciencell, United States) up to passage 6, were used in all the experiments in this study.

### 2.2 Annulus repair effect with MSCs in mechanical stimulation environment VS. in mechanical stress shied environment *in vivo*

All animal experiments were performed according to the Guidelines for Care and Use of Laboratory Animals established by the Affiliated Hospital of Integrated Traditional Chinese and Western Medicine, Nanjing University of Chinese Medicine. The stroke-bearing protocol was approved by the Committee on the Ethics of Animal Experiments of the Hospital (Ethical Lot Number: AEWC-20191010-82). The experimental procedures were approved by the hospital ethic committee. Twenty male computer-randomized SD rats (average weight 450 ± 20 g) were utilized in this study with 10% chloral hydrate anesthesia. Four caudal intervertebral discs of each rat were exposed from the ventral side for four experimental groups respectively. In brief, a 2 mm width and 2.5 mm depth lesion was punctured by a custom-made blade. Fib-T-G MSCs group: annulus lesion filled and connected by 7 μL Fib-T-G glue mixed with 1.2×10^3^ hMSCs. Fibrinogen MSCs(Fib MSCs) group: annulus lesion filled by 7 μL fibrinogen mixed with 1.2×10^3^ hMSCs and connected carefully by one micro suture (ETHICON 7-0). Un-repair control: annulus lesion without repair. Intact control: discs exposed without any intervention. All the rats were fed with standardized food and moved freely, and were all sacrificed at 5 weeks.

After the operation, the specimens were fixed with 4% neutralized formalin (BL-G001, Nanjing Senbeijia Biotechnology Co., Ltd, China), then decalcified in 10% nitric acid (USP364, CPA, France). Each segment were separated and transferred to 75% ethanol (64-17-5, Nanjing Chemical Reagent Co., Ltd, China), then embedded in paraffin, cut in the mid-sagittal plane to 5-μm thickness, and stained with hematoxylin and eosin (H&E) for cellular constituents. Histological score of disc degeneration and annulus fibrosus were evaluated based on the following four categories of degenerative changes according to previous study (Masuda et al., 2005): annulus fibrosus, border between the anulus fibrosus and nucleus pulposus, cellularity of the nucleus pulposus, and matrix of the nucleus pulposus. IVD scores range from a normal disc as 4 points to a severely degenerated disc as 12 points, and AF scores range from a normal annulus being 1 point to a more than 30% ruptured or serpentined patterned fibers having 3 points. Picrosirius red (ab150681, Abcam, United States) was stained and assessed under a polarized light microscope for lamellar structure and continuity of collagen. Diaminobenzidine (DAB, ZLI-9018, ZSGB-BIO, China) staining of the main components of annulus fibrosus collagen 1 (COL1) (1:200, ab34710, Abcam, United States), collagen 2 (COL2) (1:200, ab34712, Abcam, United States), mechanical stimulation related protein RhoA (1:100, 10749-1-AP, Proteintech, China) and ROCK1 (1:200, ab97592, Abcam, United States) were conducted according to the product instructions and viewed under a microscope (IX73, Olympus, Japan).

### 2.3 Differentiation effect of MSCs towards annulus in mechanical stimulation environment VS*.* in a static environment

The hMSCs were seeded into elastin precoated six-well BioFlex plates (Flexcell International Corporation) with 2.0×10^6^ cells/well and cultured in growth media (Dulbecco’s modified Eagle medium (DMEM, Gibco, United States) with 10% fetal bovine serum (FBS, Invitrogen, United States) and 1% penicillin-streptomycin (PS, Gibco, United States)) to reach confluence. Then cells were cultured by chondrocyte differentiation medium according to a previous study (Masuda et al., 2005; Lysdahl et al., 2013) (DMEM medium; 10% FBS; 100 nM Dexamethasone, D1756, Sigma; 50 μg/ml Ascorbic Acid-2phosphate, A4544, Sigma; 40 μg/ml L-Proline, P0380, Sigma; 1:100 ITS Supplement, 10201, Cyagen Biosciences Company; 10 ng/ml TGF-β1, 96-100-21-10, PeproTech; 1% PS), and the fresh medium was changed every 3 days. Cells were differentiated in the following two different mechanical conditions. The mechanical load group: cell cultures were loaded on the Flexercell FX 5000T cell-straining device with 3% strain and 1 HZ mechanical load stimulation for 1 h each day. The stable control group was cell cultures in a static environment.

Three cultures of each group were obtained after 7 days of differentiation. All the mediums in each well were collected to quantify the secreted sGAG and Collagens by Dimethylmethylene blue (DMMB, 341088, Sigma, United States) and Sicol assays (s1000, Biocolor, UK). Total RNA from samples was isolated using Trizol reagent (15596026, Invitrogen, United States) according to the manufacturer’s instructions. The RNA was synthesized by reverse transcription kit (E6560, NEB, United States) to cDNA, followed by quantitative real-time PCR (qPCR, ABI StepOnePlus, United States) analysis using SYBR Premix (E3003, NEB, United States) and ABI STEPONE PLUS. Gene expression of COL1A1, COL2A1, aggrecan, Mohawk, RhoA, ROCK1, and SOX9 were analyzed by qPCR. 3-phospate dehydrogenase (GAPDH) was used as a reference gene. The sequences of the genes are shown in Table 1.

**TABLE 1:** Primers sequences used for qPCR analyses.

Name		5→sequence→3	Product size	NCBI ReferenCe SequenCes (Ref Seq)
GAPDH	Sense	CCA​GAA​CAT​CAT​CCC​TGC​CT	185	NM_001256799
	Antisense	CCT​GCT​TCA​CCA​CCT​TCT​TG		
COL1A1	Sense	GTG​CTA​AAG​GTG​CCA​ATG​GT	228	NM_000088.3
	Antisense	CTC​CTC​GCT​TTC​CTT​CCT​CT		
RhoA	Sense	TCG​TTA​GTC​CAC​GGT​CTG​GT	116	NM_001313941.2
	Antisense	GCC​ATT​GCT​CAG​GCA​ACG​AA		
ROCK1	Sense	CTG​CAA​CTG​GAA​CTC​AAC​CAA​G	100	NM_005406.3
	Antisense	ATT​CTT​CTA​CCA​ATT​GCG​CTT​GC		
Aggrecan	Sense	CCT​CTG​GAC​AAC​CAG​GTA​TTA​G	97	NM_001135
	Antisense	CCA​GAT​GTT​TCT​CCA​CTC​AGA​T		
COL2A1	Sense	TCC​ACG​GAA​GGC​TCC​CAG​AA		NM_001844.5
	Antisense	CCT​GCT​ATT​GCC​CTC​TGC​CC	141	
Mohawk	Sense	CAA​GCA​AGG​ATG​ACA​CGT​ATT​G	105	NM_001242702.1
	Antisense	GGA​TGA​TGC​AGC​TGG​TAG​TT		
SOX9	Sense	GAG​CTG​AGC​AGC​GAC​GTC​AT	130	NM_000346.4
	Antisense	CGT​AGC​TGC​CCG​TGT​AGG​TG		

Another three cultures of each group were obtained after 2 weeks of differentiation. Cell cultures were soaked in 4% paraformaldehyde in PBS with pH 7.4 for 10 min at room temperature and washed with ice-cold PBS three times. Samples were incubated with PBS containing 0.5% Triton X-100 for 10 min and subsequently washed in PBS three times. Then the samples added with 3% BSA in PBS were cultured for 30 min to block the unspecific binding of the antibodies. Samples incubated in the primary antibody COL1, COL2, Aggrecan (1:200, ab36861, Abcam, United States) in PBS-1% BSA solution in a humidified chamber overnight at 4 °C. After removing the primary antibody incubation solution, the cells were washed 3 times with PBS and then coincubated with DAB staining solution at room temperature. Picro Sirius red was stained to assess the alignment of collagen. Finally, all the samples were observed by a fluorescence microscope (IX73, Olympus, Japan).

### 2.4 MSCs differentiation towards annulus tissue with transfected VS*.* non-transfected RhoA gene in a static environment

The RhoA gene was transfected to hMSCs. In brief, hMSCs of 2.0×10^6^ cells/well were plated into six-well BioFlex culture plates precoated with elastin in culture media and allowed to reach 80% confluence. Transient transfections with the plasmids encoding wild-type RhoA (Public Protein/Plasmid Library, China) and the constitutively active Q63L RhoA were carried out in the RhoA using Lipofectamine™ LTX (15338100, Invitrogen, United States). Transfected cells were allowed to recover for 12 h, then trypsinized (25200, Gibco, United States), and sorted GFP-positive cells to purify only the cells containing transgenes. Sorted cells were centrifuged (1000 rpm/min), washed (PBS as washing solution), and prepared for further study.

RhoA transfected MSCs of 2.0×10^6^ each well were plated into six-well BioFlex culture plates and cultured in a differentiation medium (corresponding to 2.3), with normal MSCs culture as control. After 7 days, three samples of each group were obtained, and gene expression of COL1, COL2, Aggrecan, Mohawk, RhoA, and R was analysed by qPCR. After 2 weeks, three samples of each group were harvested, and the matrix of COL1, COL2, and aggrecan was determined by DAB staining as above study (corresponding to 2.3).

### 2.5 MSCs differentiation towards annulus tissue with inhibited VS*.* non-inhibited RhoA gene in a mechanical stimulation environment

The hMSCs of 2.0×10^6^ each well were plated into six-well BioFlex culture plates and cultured in a differentiation medium. In mechanical load with inhibitor C3 group, 3% strain and 1 HZ mechanical load stimulation was conducted for 1 h each day, and Cell Permeable RhoA Inhibitor C3 transferase based (CT04, Cytoskeleton, United States) was added. In the mechanical load group, cells differentiated with a mechanical load but without inhibitor C3. In the control, cells differentiated without mechanical load and without inhibitor C3. After 7 days, we investigated the effect of mechanical strain on AF relative gene expression and the RhoA/ROCK1 by qPCR analysis.

### 2.6 Statistics analysis

The statistical significance for normally distributed data. Significance was indicated by letters in graphs. For gene expression data, a non-parametric distribution was assumed and presented as mean ± SD for an N ≥ 3. The data were analyzed by one-way ANOVA followed by multiple comparison tests. All tests were performed with GraphPad Prism 6.0 (GraphPad Software, La Jolla, CA, United States).

## 3 Results

### 3.1 Healing analysis of groups within mechanical stimulation and mechanical stress shielding *in vivo*

**FIGURE 2:**
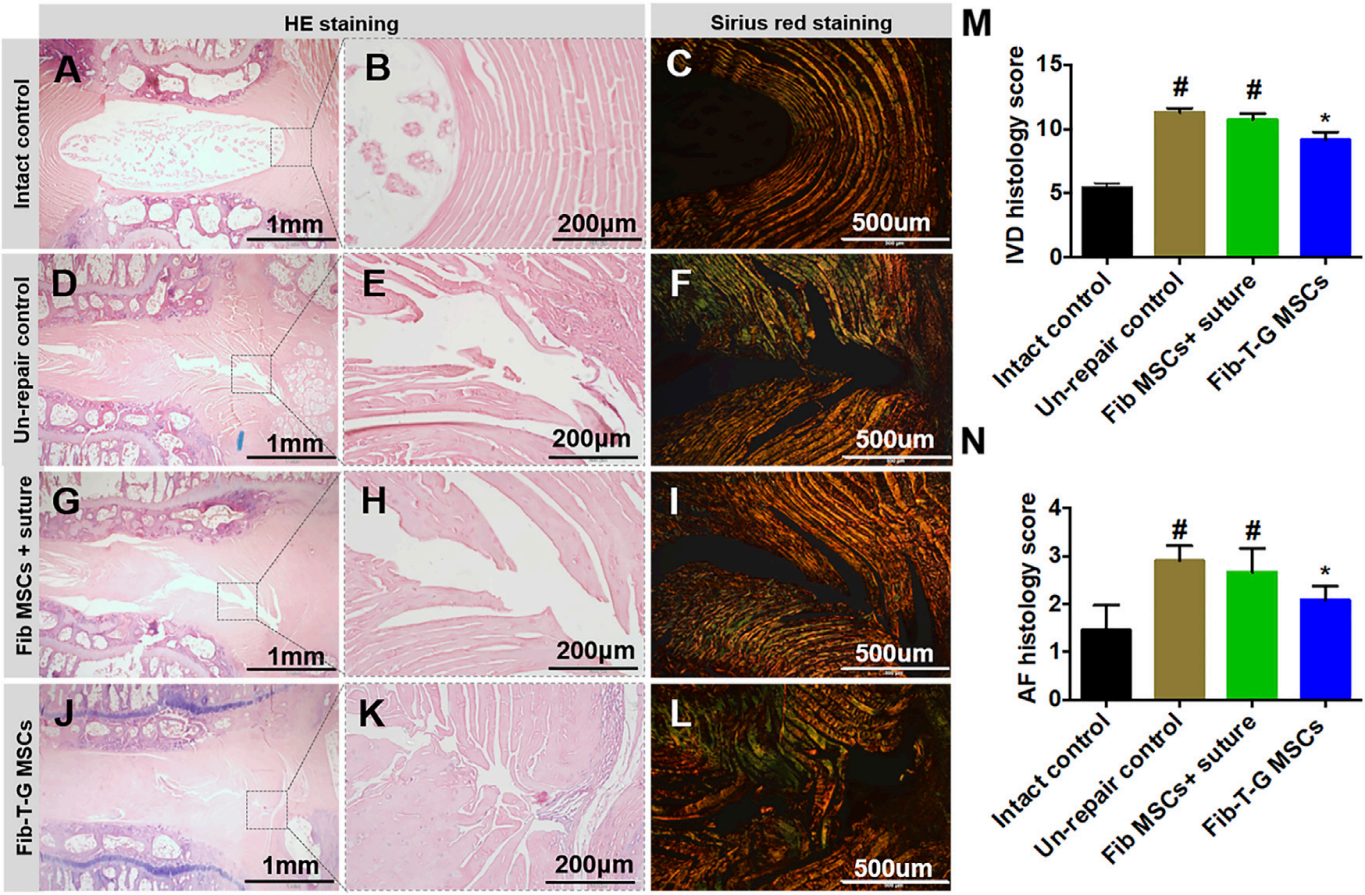
Histological assay after 5 weeks of different interventions. **(A**–**C)** The intact control disc has a loose nucleus matrix and regular annulus fibrous lamellae in HE staining, and continuous annulus lamellae are seen in Sirius red staining. **(D**–**F)** Unrepair control disc is collapsed with the severe fibrotic nucleus, obvious annulus fissure, irregular fibrous tissue in HE staining, and discontinuous annulus lamellae in Sirius red staining. **(G**–**I)** Disc repaired by Fib MSCs and suture is also collapsed with the severe fibrotic nucleus, obvious annulus fissure, irregular fibrous tissue in HE staining, and discontinuous annulus lamellae in Sirius red staining. **(J**–**L)** Disc repaired by Fib-T-G MSCs has a relatively normal structure with a slight fibrotic nucleus, slight annulus fissure in HE staining, and continuous annulus lamellae in Sirius red staining. **(M)** IVD histology score. **(N)** AF histology score. N≥3 * indicates a significant difference between the intervened discs and the unrepair control, *p* < 0.05. # indicates a significant difference between the intervened discs and the Fib-T-G MSCs group, *p* < 0.05.

**FIGURE 3:**
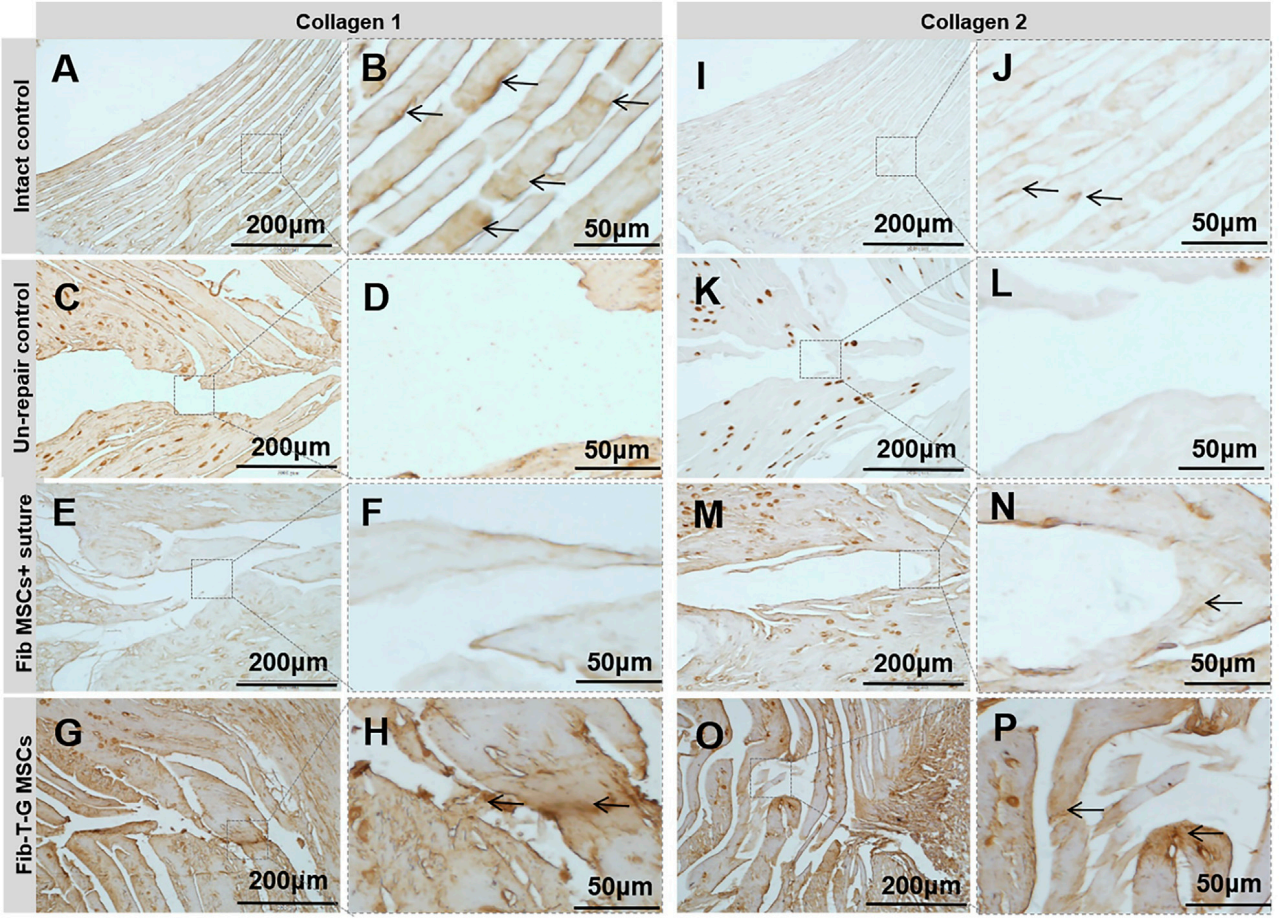
DAB staining of collagen 1 and 2 for specimens of different interventions at 5 weeks. Collage 1: **(A**, **B)** Intact control disc’s annulus has a regular, continuous structure with positive staining pointed by arrows. **(C**, **D)** Unrepair control disc’s annulus fissure is obvious with no positive staining. **(E**, **F)** Disc repaired by Fib MSCs and suture has no positive staining in the annulus fissure. **(G**, **H)** Disc repaired by Fib-T-G MSCs has a continuous structure with positive matrix staining in the annulus fissure pointed by arrows. Collagen 2: **(I**, **J)** Intact control disc’s annulus has a regular, continuous structure with positive staining pointed by arrows. **(K**, **L)** Unrepair control disc’s annulus fissure is obvious with no positive staining. **(M**, **N)** Disc repaired by Fib MSCs has some connection tissue with positive matrix staining between the fissure signed by arrows. **(O**, **P)** Disc repaired by Fib-T-G MSCs has a continuous structure with positive matrix staining in annulus fissure pointed by arrows.

**FIGURE 4:**
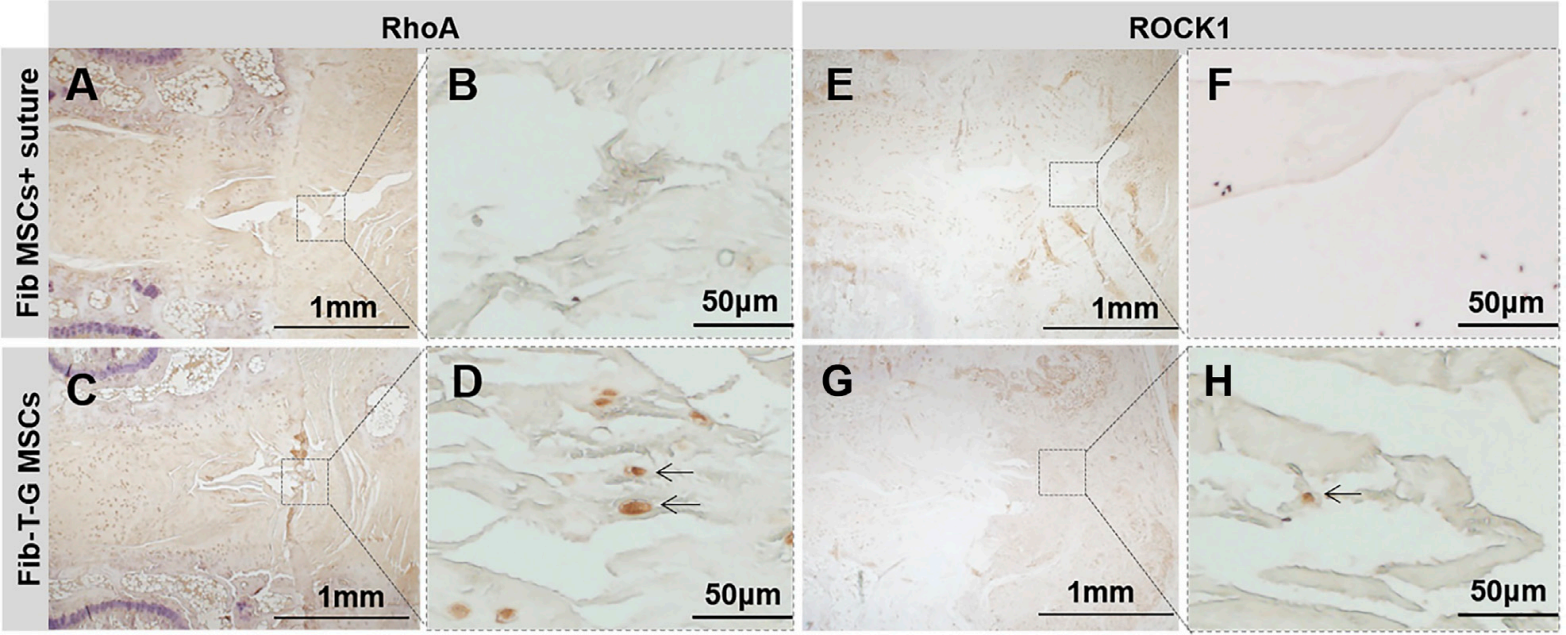
DAB staining of RhoA and ROCK1 for specimens of different interventions at 5 weeks. RhoA: **(A**, **B)** Disc repaired by Fib MSCs and suture has no positive staining in annulus fissure. **(C**, **D)** Disc repaired by Fib-T-G MSCs has a continuous structure with positive cell staining in annulus fissure pointed by arrows. ROCK1: **(E**, **F)** Disc repaired by Fib MSCs and suture has no positive staining in annulus fissure. **(G**, **H)** Disc repaired by Fib-T-G MSCs has a continuous structure with positive cell staining in annulus fissure pointed by arrows.

All the discs with lesions have degenerative signs with the fibrotic nucleus, annulus fissure, irregular fibrous tissue in HE and Sirius red staining, while the intact control has healthy loose nucleus matrix and regular annulus fibrous lamellae (Figures 2A–L). After histological disc degenerative scoring, the value 9.167 of the Fib-T-G MSCs group is significantly lower than the unrepaired control group 11.30 (*p* ≤ 0.01, and also lower than the 10.73 of the Fib MSCs group (*p* < 0.05) (Figure 2M). After histological annulus fissure degenerative scoring, the value 2.083 of the Fib-T-G MSCs group is significantly lower than the unrepaired control group 2.90 (*p* ≤ 0.01, and also lower than the 2.667 of the Fib MSCs group (*p* < 0.05). The lesion is still seen in both repaired groups but less in the Fib-T-G MSCs group at the middle sagittal histological section. The lesion area in the Fib-T-G MSCs group is apparently smaller than that in the Fib MSCs group (Figure 2N). Further, DAB staining in the repaired area of both repaired groups shows relatively more staining of collagen 1/2 (Figure 3) and RhoA/ROCK1 (Figure 4) in the Fib-T-G MSCs group, compared to the Fib MSCs group. Comprehensive results show that the Fib-T-G MSCs group with mechanical stimulation better affects repairing AF lesions.

### 3.2 Analysis of MSCs differentiation towards AF in mechanical stimulation and static environments *in vitro*

**FIGURE 5:**
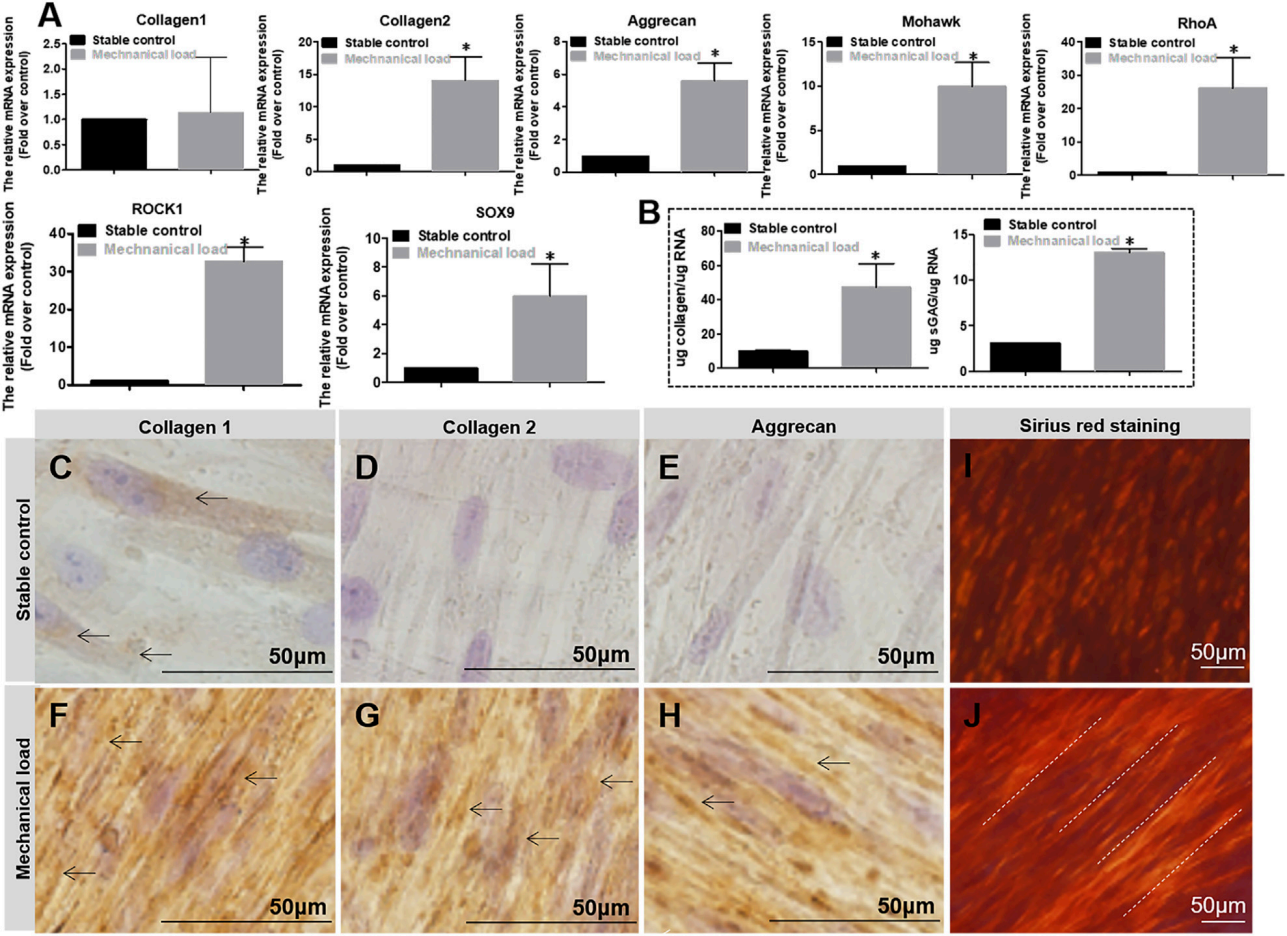
**(A)** Relative gene (Collagen1, Collagen2, Aggrecan, Mohawk, RhoA, ROCK1, SOX9) expression in different mechanical conditions after 7 days of culture. **(B)** Matrix of Collagen and Aggrecan quantification in different mechanical conditions after 7 days of culture. The expression level and quantification are expressed as mean ± SD, (n = 3). **(C**–**H)** DAB staining of Collagen 1, 2, and Aggrecan for cell culture in different mechanical conditions for 2 weeks. Cell culture differentiation in stable conditions has few positive matrix staining of Collagen 1, 2, and Aggrecan (pointed by arrows), whereas cell culture differentiation in mechanical stimulation conditions has obvious positive matrix staining of Collagen 1, 2, and Aggrecan (pointed by arrows). **(I**, **J)** Sirius red staining for cell culture in different mechanical conditions for 2 weeks. Collagen stained by Sirius red is positive but without aligned fibers seen under polarized light **(I)**; collagen stained by Sirius red is positive with obviously aligned fibers seen under polarized light (pointed by dotted lines) **(J)**. * indicates a significant difference between the different culture conditions, *p* < 0.05.

As shown in Figure 5A, after 7 days of culture, the results showed the relative fibrocartilage differentiation gene expression of COL2, aggrecan, Mohawk, RhoA, ROCK1, and SOX9 in the mechanically stimulated environment were significantly higher than those in the static environment. After 2 weeks, the secreted sGAG and collagen contents were measured by Dimethylmethylene blue and Sicol assays (Figure 5B). The results showed that the secretion amount was also significantly higher in the mechanically stimulated environment than in the static environment. In addition, mechanical stimulation promotes hMSCs differentiation with more COL1, COL2, and Aggrecan secretion observed in immunohistostaining compared to the cell culture in the static environment (Figures 5C–H). At the same time, the alignment of collagen was observed by Sirius red staining (Figures 5I, J). Arranged collagen is seen in the mechanical stimulation group under polarized light observation but not in the static group. The comprehensive results demonstrate that **the** differentiation of MSCs towards AF tissue mechanical stimulation benefits the differentiation of hMSCs towards AF tissue, along with the RhoA gene activated.

### 3.3 Mechanism analysis of mechanical stimulation environment promoting the differentiation of MSCs towards AF tissue

**FIGURE 6:**
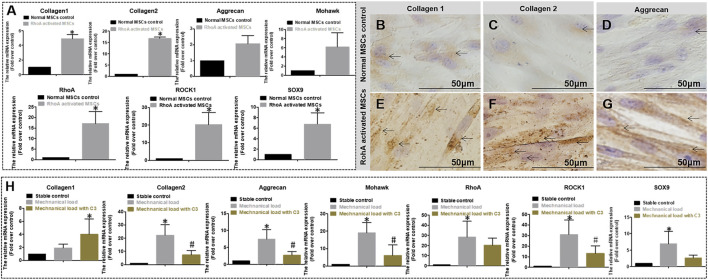
**(A)** Relative gene expression quantified by qPCR of normal and RhoA gene activated MSCs culture both in differentiation medium and without mechanical stimulation for 7 days. The expression level is expressed as mean ± SD, (n = 3) * indicates a significant difference between the different MSCs, *p* < 0.05. **(B**–**G)** DAB staining of Collagen 1, 2, and Aggrecan for two different cell cultures without mechanical stimulation for 3 weeks. **(B**–**D)** Normal MSCs differentiation has Collagen 1, 2, and Aggrecan matrix staining (pointed by arrows); **(E**–**G)** RohA gene-activated MSCs differentiation has obvious positive matrix staining of Collagen 1, 2, and Aggrecan (pointed by arrows). **(H)** Relative gene expression quantified by qPCR of MSCs differentiation without mechanical stimulation, with mechanical stimulation, or with mechanical stimulation and RhoA inhibitor C3 for 7 days. The expression level is expressed as mean ± SD, (n = 3)*indicates a significant difference compared to the cell culture without mechanical stimulation, *p* < 0.05. # indicates a significant difference compared to the cell culture with mechanical stimulation, *p* < 0.05.

As shown in Figure 6A, in the static environment, gene expression of COL1, COL2, Aggrecan, Mohawk, RhoA, ROCK1, and SOX9 in RhoA transfected hMSCs was significantly higher than that in normal non-RhoA transfected hMSCs after 7 days of culture. Relative more COL1, COL2, and aggrecan secreted by hMSCs by RhoA transfected hMSCs were observed by DAB staining (Figures 6B–G) after 2 weeks in culture. These results suggest that RhoA upregulation can promote AF-related gene expression and protein secretion. As shown in Figure 6H, in the mechanical stimulation environment, C3 Transferase inhibited RhoA signal hinders MSCs differentiation with significantly lower gene expression of collagen 2, aggrecan, Mohawk, ROCK1, SOX9 compared to the cell culture with no C3 Transferase after 7 days of culture. To sum up, without mechanical stimulation, overexpressing RhoA could promote differentiation; inhibiting RhoA reduced the differentiation effect with mechanical stimulation.

## 4 Discussion

Although AF treatment is still at an early stage (Bron et al., 2008), the necessity is increasingly recognized (Huang et al., 2018; Liu et al., 2022b). AF is a stiff ring-shaped structure withstanding complex mechanical strain (Holzapfel et al., 2004), which requires repair strategies to offer sufficient mechanical stability and potential for regenerating AF tissue. Tissues and Cells are exposed to various mechanical forces, mechanical load is very important for the health of IVD. The excessive load could damage IVD; on the other side, an appropriate load could stimulate healing. As reported, appropriate tension strain can reverse degenerated IVD, which is induced by excessive compression *in vivo* (Unglaub et al., 2006). In this study, we also find that mechanical stimulation promotes MSCs healing AF lesions *in vivo*, by means of immersing the cells in biological glue. Further *in vitro* study confirmed that certain strain forces did promote MSCs differentiation to AF-like tissue; meanwhile, RhoA gene activation was observed.

To connect the AF lesion, some stress shielding, and stress retention repair methods have been studied. However, the comparative study of these two methods has not been reported. We used biological glue and sutures to connect the lesion, representing the two mechanical environments for the differentiation of MSCs. However, the biological glue used often exhibits insufficient mechanical properties to meet the application requirements. Therefore, it is necessary to use a cross-linking agent for improvement. Genipin acts as a natural cross-linking agent that can connect tissues or gels at the molecular level. In addition, genipin is also an iridoid compound with multiple active groups, such as hydroxyl and carboxyl groups, which can spontaneously react with amino acids to form blue pigments and maintain a good degree of cross-linking. More importantly, compared to other commonly used cross-linking agents, such as glutaraldehyde (GA), carbodiimide, epoxy compounds, etc., genipin is considered to be the least toxic cross-linking agent, which can effectively reduce the immunogenicity of xenogeic matrices (Oryan et al., 2018). In the degenerative intervertebral disc, genipin crosslinked gel is considered degradate more slowly than gel without genipin (Likhitpanichkul et al., 2014; Likhitpanichkul et al., 2015), and the use of genipin crosslinking has been shown to alter several mechanical parameters (Fujii et al., 2020) and may be advantageous in treating disc pain (Guterl et al., 2013; Guterl et al., 2014; Peng et al., 2020) and disc degeneration (Yu et al., 2021). Thus, we tried genipin and tested Fib-T-G (F140 G6) gel as the best consistency for biocompatibility and adhesive strength; afterward, we applied this biological glue to repair the caudal AF slit injury of rats. It was demonstrated that the Fib-T-G gel helps treat the AF fissure and prevent disc degeneration, more than that, crosslinked sticky gel (Fib-T-G MSCs gel) got better repair results than the unstuck gel (Fib MSCs gel). The result stresses the importance of the mechano-transferred gel and the mechanical stress for the differentiation of the embedded MSCs, which is consistent with published studies (Delaine-Smith and Reilly, 2011). The reason might be that the injected genipin crosslinked hydrogel deforms almost entirely uniformly and transmit physiology tension; thus, the strain force resulting from the repaired disc would be relatively homogeneous on each stem cell throughout the hydrogel scaffold so as to produce continuous ECM to bridge cells and induce the cell differentiation for AF like tissue.

This result predicts a better repair effect of our method in the human body. In general, biological glue provided the initial stabilization of the lesion and stress transmission to the inside MSCs. Along with glue degradation, newly generated tissue gradually filled the lesion. Forces provide crucial signals to inform cell behavior, to know the intrinsic mechanism, previous studies have implied that the RhoA/ROCK1 signaling pathway may be an important molecular mechanism for differentiating MSCs into skeletal muscle tissue in response to stress stimuli (Heo et al., 2016). Whether mechanical stimulation also regulates the expression of annulus-like genes and proteins in MSCs differentiation is unclear. In order to further explore how the mechanical microenvironment promotes MSCs healing AF, *in vitro* mechanical stimulation was used to simulate the *in vivo* study. Our results demonstrated that genipin crosslinked hydrogels show off better results of disc healing in the repaired zone than the Fibrinogen with suture group in rats, as well as support mechanical strain up-regulated AF tissue markers Mohawk, SOX-9 and ECM markers COL2 and aggrecan after application in MSCs, exhibit trends that are consistent with those reported with regard to fibrous tissues (Baker et al., 2011; Elsaadany et al., 2017). The above study strengthens the evidence that the physical microenvironment plays a vital role in cell differentiation (Raabe et al., 2013; Elsaadany et al., 2017) and extracellular matrix remodeling (Higuchi et al., 2013; Lee et al., 2016). Our study addresses the performance of strain-related mechanotransduction in AF fissure repair of MSCs and explores the mechanical mechanism, the different mechanical strains, and the frequency regulating differentiation of stem cells we refer to previous studies (Elsaadany et al., 2017) and take a preliminary verification.

There are stress-sensitive receptors on the cell surface, which can transmit the induced mechanical signals into the cells through special molecular channels on the cell surface to achieve force-chemical conversion, thereby regulating the physiological functions of cells (Dupont and Wickström, 2022). Some studies have shown that pressure stimulation of the IVD cartilage endplate can activate the RhoA/ROCK1 signaling pathway, leading to changes in gene expression of COL2, Proteoglycan, and SOX9 (Xu et al., 2016). Recent studies have also shown that the RhoA/ROCK signaling pathway is an important molecular mechanism by which MSCs respond to stress stimuli and regulate their differentiation into skeletal muscle tissue (McBeath et al., 2004; Amano et al., 2010; Lessey et al., 2012; Heo et al., 2016). Our study investigated the downstream signaling pathway of RhoA, the expression of ROCK1 and SOX9 were increased in response to mechanical strain. To elucidate whether RhoA controlled strain-induced differentiation, we take overexpression of RhoA, which results in the recruitment of ROCK1 and enhanced differentiation. Evidence also from the other side showed reduced differentiation efficiency when inhibiting RhoA in a mechanical stimulation environment.

There are some limitations in our study. Firstly, rat tail caudal AF fissure models experience much less tension strain and compression force than humans. This does not affect the significance of this study since the healing principle should be similar to humans. And with the quality of adhesion and compatibility of glue improvement by different crosslinking methods (Dare et al., 2009; Cruz et al., 2017; Moriguchi et al., 2018), it will eventually reach the standard for human AF repair. Secondly, although locking cells in the damaged space is the advantage of using biological glue for AF repair, unlike in the control group, where the cells may cause leakage out in the non-stick fibrinogen solution, the resulting different amount of MSCs in each group may affect the comparison study in the rat model. And this is one of the reasons why we need to conduct accurate *in vitro* cell experiments. Thirdly, the exact mechanism of this repair strategy remains to be further verified. Alongside the RhoA/ROCK1 signaling, other pathways may be responsible for mechanotransduction regulating fibrochondrogenic differentiation of MSCs (Wang et al., 2013; Xin et al., 2022) and are worth deep research and further elaboration.

## 5 Conclusion

Mechanical stimulation promotes MSCs healing of the lesion of AF in the rat model by immersing the cells in the Fib-T-G glue to connect the lesion. Mechanical stimulation is vital for AF tissue regeneration by regulating the differentiation of MSCs, possibly *via* RhoA/ROCK1 signaling.

## Data Availability

The original contributions presented in the study are included in the article/Supplementary Material, further inquiries can be directed to the corresponding authors.
