# Supplementary material for: Mechanical stimulation promotes MSCs healing the lesion of intervertebral disc annulus fibrosus
Source: Front Bioeng Biotechnol. 2023 Feb 10;11:1137199. doi: 10.3389/fbioe.2023.1137199 (PMC9950411; doi:10.3389/fbioe.2023.1137199)
Supplement: Supplementary file 1 [file DataSheet1.docx]

**Mechanical stimulation promotes MSCs healing the lesion of intervertebral disc annulus fibrosus**

Rongrong Deng^1^, Ran Kang^1,2^*, Xiaoyu Jin^1,3^, Zihan Wang^1^, Xin Liu^1^*, Qing Wang^4^, Lin Xie^1^*

________________________________________________________________________________

*^1^* *Third School of Clinical Medicine, Nanjing University of Chinese Medicine, Nanjing, Jiangsu Province 210028 P.R. China.*

*^2^Department of Orthopedics, Nanjing Lishui Hospital of Traditional Chinese Medicine, Nanjing, Jiangsu*

*Province 210028 P.R. China.*

*^3^Department of Sports Medicine and Adult Reconstructive Surgery, Nanjing Drum Tower Hospital, Nanjing, Jiangsu Province 210008 P.R. China.*

*^4^School of Nursing, Nanjing University of Chinese Medicine, Nanjing, Jiangsu Province 210046 P.R. China.*

***Correspondence:** [kangran@njucm.edu.cn](mailto:kangran@njucm.edu.cn) (Ran Kang), [liuxin@njucm.edu.cn](mailto:liuxin@njucm.edu.cn) (Xin Liu),

[xielin@njucm.edu.cn](mailto:xielin@njucm.edu.cn) (Lin Xie)

**Supplemental Methods**

## Fibrinogen-Thrombin-Genipin (Fib-T-G) gel cytotoxicity test *in vitro*

Gels were created in the wells of 48-well plates. Into each well, fibrinogen (35 μl/well) and genipin were pipetted, immediately Thrombin (7 μl/well) was added, and finally obtained Fib-T-G gel fabrication (Fib-T-G3, Fib-T-G6, Fib-T-G8) with the concentration of genipin were 3, 6, 8 mg/ml respectively (n = 6). After 24 hours of incubation, 2500 hMSCs in 200 ul Mesenchymal Stem Cell medium per well were implanted on the surface of the fabrication of the Fib-T-G gel. The mixed biology gel was fed with the fresh growth medium every 2 days, and cell viability was quantified by XTT Kit (11465015001, Roche, Switzerland) on days 1, 3, and 7. Finally, On day 7, after rinsing by PBS, the biology gel was incubated with DAPI dye solution (F6057, Sigma, USA) for 15 min at 37 °C. Cell proliferation was stained and visualized under a fluorescence microscope (IX73, Olympus, Japan), followed by quantitative analysis by ImageJ software. From each experimental well, five fields of view were captured and counted. Each group's total DNA and RNA were extracted with Universal Genomic DNA Kit (CW2298S, CWBIO, China) and Trizol (15596026, Invitrogen, USA), respectively, and measured by Microplate Reader on day 7.

**Fibrinogen-Thrombin-Genipin (Fib-T-G) gels mechanical property measurement**

Fib-T-G gels fabrication (F140G0, F140G3, F140G6, F140G8) were prepared, samples (n = 24) made by rubberized fabric got 10 mm×10 mm overlap area (Figure S2A). To make sure the products croslinking sufficient, before lap testing ,the samples should be put in the PBS bath for 24 hours. Based on our previous research[18], failure tests were carried out by the mechanical testing system (Bionix 370,MTS Systems Corporation, Eden Prairie, MN) with gripping the overhang on either end of the fabric and extending to failure at 0.1 mm per second (Figure S2B). Interfacial strength was determined from the maximum force normalized to overlap area.

**Supplemental Results**

***In vitro* cytotoxicity evaluation and Mechanical analysis of Fibrinogen-Thrombin-Genipin (Fib-T-G) gels**

The final purpose of the prepared injectable Fib-T-G gels is the repair of annulus fibrosus. Therefore, MSCs, as the most important pro-annulus repair differentiated cells, were used to evaluate the biocompatibility of the gels. MSCs were incubated with Fib-T-G gels with different concentrations of genipin for 1 d, 3 d, and 7 d. Then the effect of each concentration of gels on cell viability was detected by the XTT kit. As shown in Figure S1, XTT assay result showed increasement in cell number between day 1 and day 7 was statistically significant (p < 0.05) for Fib-T-G3 gels and Fib-T-G6 glues. To further verify the biocompatibility of gels with different genipin concentrations, cells on day 7 were stained with DAPI to observe cell proliferation, quantified, and averaged across 3 fields. Finally, immunofluorescence images and statistical analysis showed that Fib-T-G6 and Fib-T-G3 gels also showed better biocompatibility than Fib-T-G8 gels (Figure S2). In addition, we also detected the total DNA and RNA content of each sample group. As shown in Figure S3, the total DNA content showed the same results as above, while the total RNA content of the Fib-T-G6 gel group was much larger than that of the Fib-T-G3 and Fib-T-G8 groups.

Injectable Fib-T-G gels used to repair annulus fibrosus inevitably need to withstand pressure from surrounding tissues, and insufficient pressure is prone to rupture, resulting in poor repair results. Therefore, the as-prepared Fib-T-G gels should have good mechanical properties. As shown in Figure S4C, Fib-T-G0 showed a failure force of 14.17±3.25%, showing the lowest mechanical properties of all samples. The failure forces of Fib-T-G3, Fib-T-G6, and Fib-T-G8 were 18.25±1.09%, 21±1.58%, 20.83±2.41%, respectively, which were significantly higher than those of the Fib-T-G0 group. Among them, Fib-T-G6 exhibits the highest failure force, indicating that it has the best mechanical properties. The mode of specimen failure was equally distributed between slippage of the gel-tissue interface and fracture of the gel itself. Therefore, combined with the above biocompatibility test results and considering the mechanical strength, Fib-T--G6 was screened out as the best gel parameters for subsequent animal and cell experiments and collectively referred to as "Fib-T-G".

**Supplemental Figures**


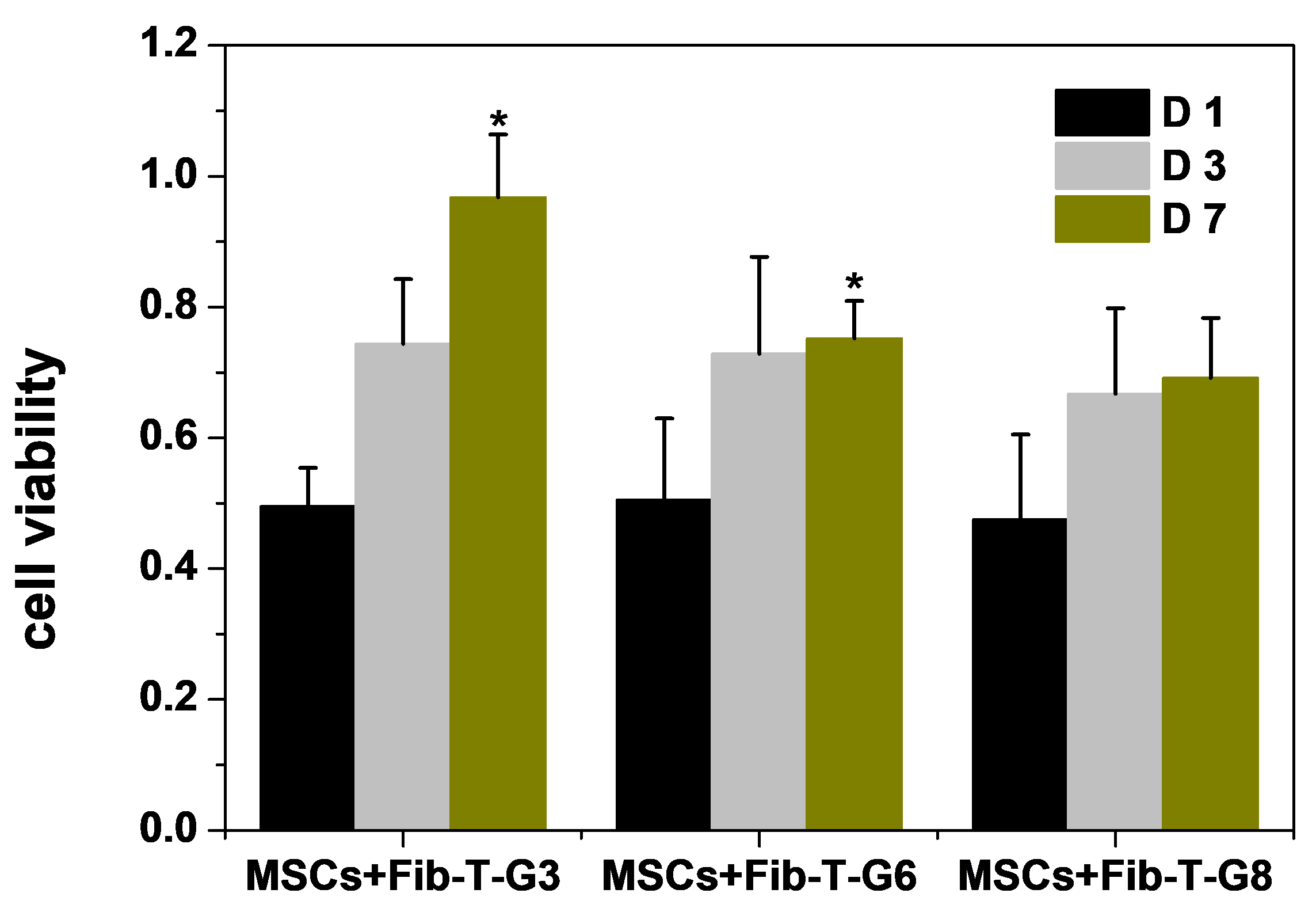


**FIGURE S1.** The XTT assay results of MSCs on Fib-T-G gels with the different concentrations of genipin, N ≥ 3. . * Indicates a significant difference between the D 7 and D 1, p < 0.05.

**FIGURE S2.** Representative DAPI staining fluorescence images (**A**) and corresponding statistics (**B**) of Fib-T-G gels with the different concentrations of genipin co-cultured with MSCs after 7 days, N ≥ 3. * Indicates a significant difference between the Fib-T-G6 and Fib-T-G3, p < 0.05. # Indicates a significant difference between the Fib-T-G8 and Fib-T-G3, p < 0.05.


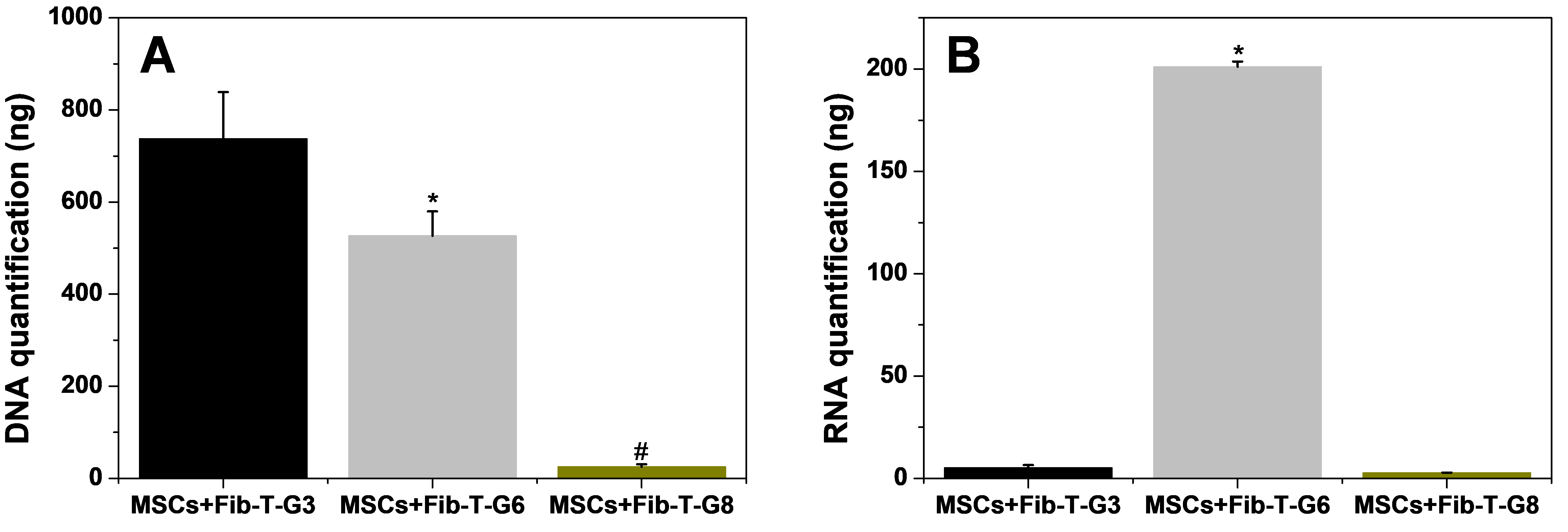


**FIGURE S3.** The total DNA (**A**) and RNA (**B**) of MSCs on Fib-T-G gels with the different concentrations of genipin after 7 days, N ≥ 3. * Indicates a significant difference between the Fib-T-G6 and Fib-T-G3, p < 0.05. # Indicates a significant difference between the Fib-T-G8 and Fib-T-G3, p < 0.05.


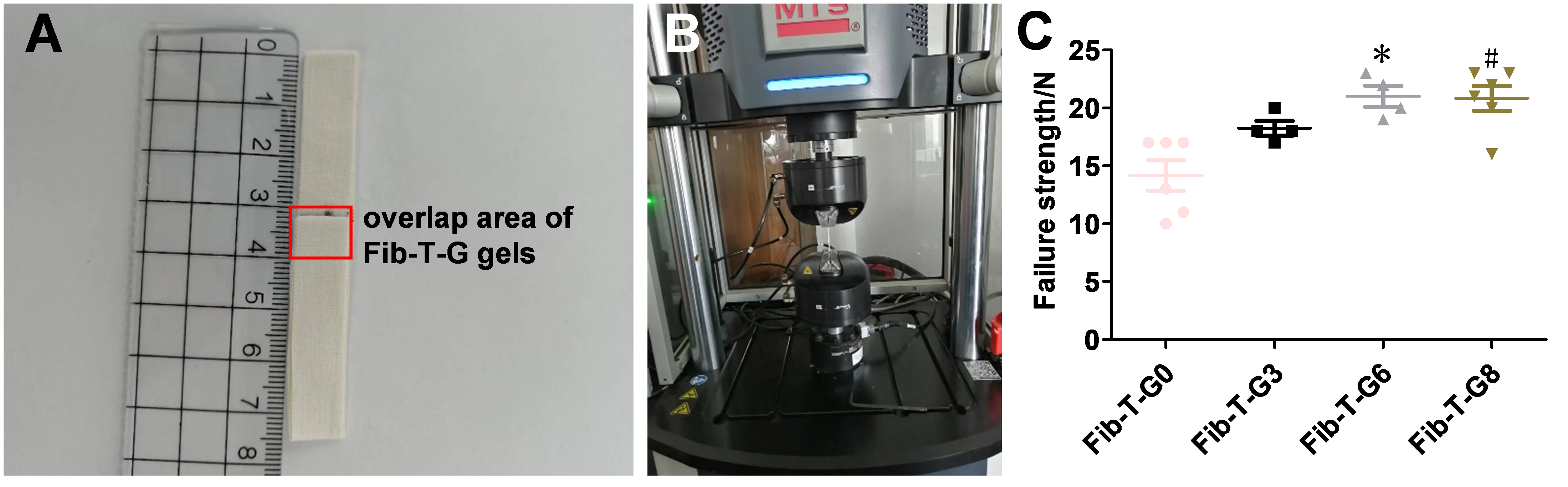


**FIGURE S4.** . (**A**) Representative pictures of samples made of rubberized fabrics; (**B**) Schematic diagram of the mechanical strength test process. (**C**) The mechanical strength of Fib-T-G gels with the different concentrations of genipin, N ≥ 3. * Indicates a significant difference between the Fib-T-G6 and Fib-T-G0, p < 0.05. # Indicates a significant difference between the Fib-T-G8 and Fib-T-G0, p < 0.05.
